# Differential effects of stress exposure via two types of restraint apparatuses on behavior and plasma corticosterone level in inbred male BALB/cAJcl mice

**DOI:** 10.1002/npr2.12093

**Published:** 2019-12-23

**Authors:** Hirotaka Shoji, Tsuyoshi Miyakawa

**Affiliations:** ^1^ Division of Systems Medical Science Institute for Comprehensive Medical Science Fujita Health University Toyoake Japan

**Keywords:** BALB/cA, depression, inbred strain, mouse model, repeated restraint stress

## Abstract

**Aims:**

Restraint stress is one of the most widely used experimental methods for generating rodent models of stress‐induced neuropsychiatric disorders, such as depression and anxiety. Although various types of restraint apparatuses have been used to expose animals to stress, the magnitudes of the effects of stress exposure via different types of restraint apparatuses on physiology and behavior have not been compared in the same environment. Here, we investigated the effects of stress exposure via two types of restraint apparatuses on body weight, locomotor activity, anxiety‐ and depression‐related behaviors, and plasma corticosterone levels in mice.

**Methods:**

Adult male BALB/cAJcl mice were restrained by placing them in either a well‐ventilated plastic conical tube or a tapered plastic film envelope for 6 hours per day for 10 or 21 consecutive days. Mice were weighed during and after the stress period and were subjected to a battery of behavioral tests, including light/dark transition, open field, elevated plus maze, Porsolt forced swim, tail suspension, and sucrose preference tests, starting on the day after the last stress session. Plasma corticosterone levels were measured in another cohort of mice on the 1st and the 21st stress sessions and after the Porsolt forced swim test.

**Results:**

Exposure to repeated stress via the two above mentioned types of restraint apparatuses caused body weight loss, heightened locomotor activity, altered immobility during forced swim, and increased plasma corticosterone levels, and some of these results differed between the restraint stress protocols. Film‐restraint–stressed mice had significantly lower body weights than tube‐restraint–stressed mice. Film‐restraint–stressed mice exhibited significantly higher or lower immobility during forced swim than tube‐restraint–stressed mice, depending on the test time. Additionally, the stress‐induced increase in plasma corticosterone levels was found to be higher in film‐restraint–stressed mice than in tube‐restraint–stressed mice.

**Conclusion:**

Our results indicate that film‐restraint stress has more pronounced effects on body weight, depression‐related behavior, and corticosterone response than tube‐restraint stress in mice. These findings may help guide which restraint stress procedures to use, depending on the objectives of a given study, in generating animal models of stress‐induced neuropsychiatric disorders.

## Abstract 2

This study investigated the effects of stress exposure via two types of restraint apparatuses, a well‐ventilated plastic conical tube and a tapered plastic film envelope, on body weights, behaviors, and plasma corticosterone levels in male BALB/cAJcl mice. Film‐restraint stress had more pronounced effects on body weight, depression‐related behavior, and corticosterone response than tube‐restraint stress, which may help guide which restraint stress procedures to use in generating animal models of stress‐induced disorders.

## Graphical abstract

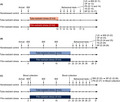

## 1 INTRODUCTION

Stress that we experience in our daily lives initiates a variety of physiological processes of the central and peripheral systems, typically leading to neuroendocrine responses, such as glucocorticoid (cortisol in humans and corticosterone in rodents) secretion, through the activation of the hypothalamic‐pituitary‐adrenal (HPA) axis.^1^, ^2^ The physiological responses to stress are necessary for brain homeostasis and adaptation to stress.^1^, ^3^ However, exaggerated and prolonged neuroendocrine responses to chronic excessive stress are considered to be associated with the pathogenesis of neuropsychiatric disorders, including depression and posttraumatic stress disorder.^1^, ^2^ In rodent models of chronic stress, physical restraint, which is one of the most widely used methods of stress exposure, has been performed by placing mice into various restraint apparatuses, including a wire‐mesh restrainer, wire‐mesh gauze, wire‐mesh cage, rigid plastic tube, or plastic film envelope (DecapiCone), for several minutes to several hours (in most cases, for 2‐6 hours) each day for a number of weeks (typically 1‐3 weeks) (for review, see Refs 4, 5, 6). Acute and chronic exposure to restraint stress has been reported to induce multiple physiological and behavioral changes, such as elevated levels of glucocorticoids, body weight loss, altered locomotor activity, heightened anxiety‐like and depression‐related behaviors, decreased sucrose preference (anhedonia), and learning and memory impairments^7^, ^8^, ^9^, ^10^ (for review see Ref. 6). Various types of restraint apparatuses have been used in different laboratories to understand stress responses and to study animal models of stress‐induced neuropsychiatric disorders, but little attention has been paid to the possibility that different types of restraint apparatuses could result in differential magnitudes of effects on behavioral and glucocorticoid responses to stress.

In this study, we compared the effects of repeated restraint stress in two types of restraint apparatuses, a plastic conical tube (well‐ventilated 50‐mL tube) and a tapered plastic film envelope (DecapiCone), which have been widely used to produce an animal model of stress‐related disorders,^6^ on endocrine and behavioral responses in an inbred strain of BALB/cAJcl adult male mice. Mice were restrained for 6 hours per day for 10 or 21 consecutive days. Nonstressed control mice were left undisturbed in their home cage. Their body weights were measured during and after the stress period. The animals were subjected to a battery of behavioral tests, including the light/dark transition test, open field test, Porsolt forced swim test, tail suspension test, and sucrose preference test to assess locomotor activity, anxiety‐like behavior, and depression‐related behavior, starting the day after the final stress session. Plasma corticosterone levels were measured on the 1st and 21st repeated stress sessions and after the forced swim test.

## 2 METHODS

### 2.1 Animals

Seven‐week‐old naïve male BALB/cAJcl mice were purchased from CLEA Japan, Inc. The mice were transported from the Fuji Breeding Center of CLEA Japan, Inc. to our animal facility. Three independent cohorts of 70‐73 mice were used. After arrival, mice were group‐housed (two to four per cage) in a plastic cage (250 × 182 × 139 mm), with paper chips for bedding (Paper Clean; Japan SLC, Inc.), covered with a stainless steel grid lid. The mice were acclimated for at least 1 week before the experiments started (8‐14 weeks of age at the start). Rooms were maintained on a 12‐hour light/dark cycle (lights on at 7:00 am) and at 23 ± 2°C. The mice were provided with filtered tap water and food pellets (CRF‐1; Oriental Yeast Co., Ltd.) ad libitum, although mice assigned to the stress conditions described below did not have access to water or food for the duration of stress exposure. All of the experimental procedures were approved by the Institutional Animal Care and Use Committee of Fujita Health University.

### 2.2 Stress exposure

**Figure 1:**
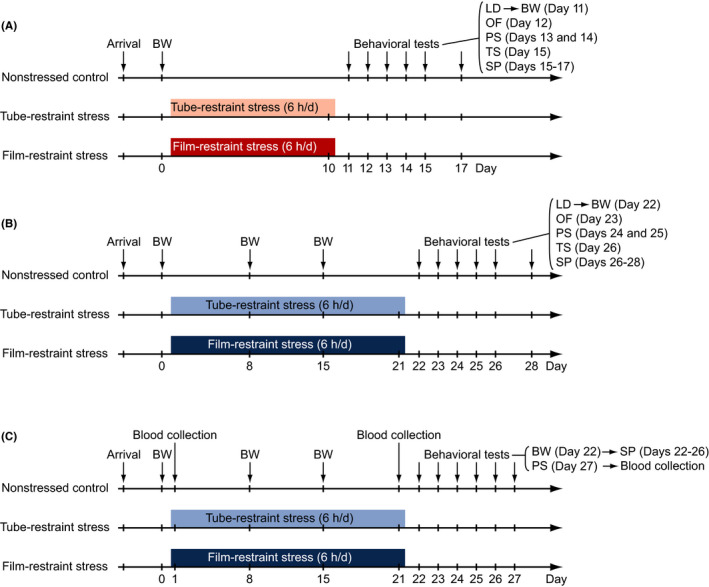
Schematic diagram of experimental procedures. Experimental procedures for the three different cohorts of mice exposed to restraint stress for 10 d (A) or for 21 d (B, C) are illustrated. Mice in the stress groups were exposed to restraint stress daily for 6 h each day by being placed in either a well‐ventilated 50 mL polypropylene conical tube (tube‐restraint stress group; Tube) or a tapered plastic film tube that was twisted and squeezed at the large, open end (film‐restraint stress group; Film). Nonstressed control mice were left undisturbed until the start of behavioral testing, except for cage cleaning, weighing (BW), or blood collection (nonstressed control group; Con). After the stress session, mice in each group were subjected to a battery of behavioral tests, including the light/dark transition test (LD), open field test (OF), Porsolt forced swim test (PS), tail suspension test (TS), and sucrose preference test (SP), in the order presented in this figure

Each cage of mice was divided into the following three groups: nonstressed control (Con), tube‐restraint stress (Tube), and film‐restraint stress (Film) groups. Mice in the two stress groups were exposed to restraint stress for 6 hours per day. Stress sessions started between 10 am and 12 pm. Mice in the tube‐restraint stress group were placed in a well‐ventilated 50 mL polypropylene conical tube (114.4 mm long, 29.1 mm outer diameter; Corning Inc.), and a quarter of a paper towel (Kim Towel folding in four, 380 × 330 mm; Nippon Paper Crecia Co., Ltd.) was placed in the tube to fill the space between the mouse and the tube cap. Mice in the film‐restraint stress group were put into a tapered plastic film envelope (Mouse DecapiCone, MDC‐200; Braintree Scientific, Inc.), which was twisted and squeezed at the large, open end. The restrained mice were placed in their home cage during the 6‐hour stress session. The stress procedure was performed in a sound‐attenuated room adjacent to the housing room. Mice in the nonstressed control group were left undisturbed in their home cages, except when measuring body weight and regular cage cleanings. The control mice were allowed free access to food and water during the 6‐hour stress session. Mice in the first cohort were restrained for 10 consecutive days. The other two cohorts of mice received stress sessions for 21 consecutive days. The first session of stress exposure was designated Day 1. The first cohort (Con, n = 24; Tube, n = 24; Film, n = 22) and second cohort (Con, n = 24; Tube, n = 24; Film, n = 25) of mice underwent body weight measurements and a battery of behavioral tests (for the first cohort, Figure 1A; for the second cohort, Figure 1B). The third cohort of mice (Con, n = 23; Tube, n = 24; Film, n = 25) underwent plasma corticosterone level measurement and body weight measurement during and after stress exposure, and approximately half of the mice were subjected to behavioral tests (Figure 1C; for details, see Section 2.6).

### 2.3 Body weight measurement

Mice in the first cohort were weighed before the first session of stress exposure and one day after the last session of 10‐day stress exposure. Mice in the second and third cohorts were weighed weekly; specifically, body weight measurements were performed before the first session of stress exposure (Day 0), before the start of the stress sessions on Day 8 and Day 15, after behavioral testing on Day 22, and on Days 29‐32. Percent body weight was calculated based on the body weight on Day 0 (body weight (%) = 100 × [body weight (g)/body weight (g) on Day 0]).

### 2.4 Behavioral tests

The first and second cohorts of mice were subjected to a battery of behavioral tests in the following order starting with those considered to be least stressful: light/dark transition test (for the first cohort, Day 11; for the second cohort, Day 22), open field test (for the first cohort, Day 12; for the second cohort, Day 23), Porsolt forced swim test (1 trial/d for 2 days: for the first cohort, Days 13 and 14; for the second cohort, Days 24 and 25), tail suspension test (for the first cohort, Day 15; for the second cohort, Day 26), and sucrose preference test (across 2 days: for the first cohort, Day 15‐17; for the second cohort, Day 26‐28). A portion of the third cohort of mice was tested in the sucrose preference test (across 4 days: Days 22‐26) and the Porsolt forced swim test (1 trial/d for 1 day: Day 27) following the 21‐day stress period. After each test, the floors and walls of the testing apparatuses were cleaned with water with hypochlorous acid to eliminate any olfactory cues.

#### 2.4.1 Light/dark transition test

The light/dark transition test, originally developed by Crawley and Goodwin,^11^ was performed as previously described.^12^ The apparatus consisted of a cage (21 × 42 × 25 cm) divided into two sections of equal size by a partition with a door (O’Hara & Co.) One chamber consisted of white plastic walls and was brightly illuminated (390 lux) by lights attached above the ceiling of the chamber. The other chamber had black plastic walls and was dark (2 lux). Both chambers had a gray plastic floor. Mice were placed into the dark chamber and were allowed to move freely between the two chambers for 10 minutes with the door open. The distance traveled (cm), total number of transitions, latency to first enter the light chamber (seconds), and time spent in the light chamber (seconds) were recorded automatically using the ImageLD program (see Section 2.5).

#### 2.4.2 Open field test

The open field test was performed in the open field apparatus with the VersaMax Animal Activity Monitoring System (40 × 40 × 30 cm; Accuscan Instruments), in which the center area was illuminated to 100 lux by lights attached above the ceiling. The center area was defined as a 25 cm × 25 cm area. Each mouse was placed in one corner of an open field. Their behaviors, including the total distance traveled (cm), vertical activity (rearing measured by counting the number of photobeam interruptions), time spent in the center area (seconds), and stereotypic counts (beam‐break counts for stereotyped behaviors), were automatically recorded using an activity monitoring system for the entire 30‐minute period after mice were placed in the apparatus. The behavioral data were analyzed in 5‐minute blocks.

#### 2.4.3 Porsolt forced swim test

The Porsolt forced swim test, originally developed by Porsolt et al,^13^ was performed to assess depression‐related behavior. Mice were placed into a Plexiglas cylinder (20 cm height × 11.4 cm inner diameter; O’Hara & Co.) filled with water (21‐23°C) up to a height of 8 cm for 10 minutes. The percentage of immobility time was recorded automatically using the ImagePS/TS program (for details, see Refs 14, 15). The first and second cohorts of mice were given one test session per day for two consecutive days, following the original procedure by Porsolt et al.^16^, ^17^ The third cohort of mice was tested in one test session, and immediately after the session, their blood was collected to assess endocrine stress response to novel stimuli, that is, swim stress, after repeated restraint stress (for details, see Section 2.6).

#### 2.4.4 Tail suspension test

The tail suspension test was used to evaluate depression‐related behavior.^18^ Mice were suspended 30 cm above the floor in a visually isolated area by adhesive tape placed approximately 1 cm from the tip of the tail. Immobility was recorded for a 10‐minute test period. Immobility time was measured automatically using the ImagePS/TS program (for details, see Refs 14, 15).

#### 2.4.5 Sucrose preference test

In the first and second cohorts of mice, after the tail suspension test, mice were singly housed in a plastic cage (250 × 182 × 139 mm) with new paper chips for bedding and were subjected to a two‐bottle choice test, in which mice were provided with a bottle containing water and a second bottle containing 1% sucrose solution, with the left/right positions of the bottles counterbalanced across groups of animals. Bottles were weighed prior to testing and then again 2 days (approximately 48 hours) after the start of the test. The third cohort of mice, one day after 21‐day stress exposure, was singly housed and subjected to a 1% sucrose preference test for 4 days, during which the position of two bottles was changed every 24 hours. Sucrose preference during the first 2 days and the last 2 days of the test in the third cohort of mice was evaluated in the same manner as in the first and second cohorts of mice. In each cohort of mice, sucrose preference during the 2‐day test session was calculated as the percentage of sucrose preference = 100 × [(sucrose intake during the 2‐day session)/(sucrose intake during the 2‐day session + water intake during the 2‐day session)].

### 2.5 Image analysis

For the light/dark transition, Porsolt forced swim, and tail suspension tests, image analysis programs (ImageLD/PS/TS) were used to automatically analyze mouse behaviors. These programs, based on the public domain ImageJ program (developed by Wayne Rasband at the National Institute of Mental Health), were developed and modified for each test by Tsuyoshi Miyakawa. The ImageLD program can be freely downloaded from the website of the “Mouse Phenotype Database” (http://www.mouse-phenotype.org/).

### 2.6 Plasma corticosterone measurement

The third cohort of mice (Con, n = 23; Tube, n = 24; Film, n = 25) was used for plasma corticosterone (CORT) measurement. These groups were further randomly divided into two subgroups to collect blood at different time points during the stress procedure (for a schematic diagram of blood collection, see Figure 4A,B). In one of the subgroups (Figure 4A), on Days 1 and 21 of the repeated stress procedure, tube‐ and film‐restraint–stressed mice (Tube, n = 12; Film, n = 13) were released from the restraint apparatus 30 minutes after the onset of stress exposure, and then, approximately 0.1 mL blood was taken from the facial vein or submandibular vein using Goldenrod Animal Lancet (MEDIpoint, Inc.). The stressed mice were again placed into their restraint apparatuses to receive restraint stress for the remaining 5.5 hours. This procedure took approximately 2 minutes to complete. One hour after the end of the 6‐hour stress session, blood was again collected. In the nonstressed control mice (Con, n = 11), blood collections were carried out at the same time as those in the stressed mice. Similarly, in another subgroup of mice (Con, n = 12; Tube, n = 12; Film, n = 12), blood was collected at time points before and after the 6‐hour stress session on Days 1 and 21 (Figure 4B).

To further examine the effect of different types of stress on plasma CORT levels, approximately half of each subgroup was behaviorally tested. One half of the mice in each stress group were subjected to the forced swim test for 10 minutes in the same manner as described above. The behavioral testing was conducted between 10:30 and 13:00 on Day 27. Immediately after exposure to swim stress, blood samples were similarly obtained (Swim stress condition: n = 6 in Con; n = 6 in Tube; n = 8 in Film). At the same time, on the same day, the remaining half of the mice were taken from their home cage, and blood collection was performed (no stress condition: n = 5 in Con; n = 6 in Tube; n = 8 in Film) (Figure 4C).

Blood was collected into tubes containing 10 units of sodium heparin (Wako Pure Chemical Industries Ltd.), placed on ice and immediately centrifuged at 3000 *g* for 10 minutes at 4°C. Supernatants were collected and stored at −80°C until measurement. Plasma CORT concentrations were determined using a correlate‐enzyme immunoassay kit (Assay Designs Inc.) according to the manufacturer's protocol.

### 2.7 Statistical analysis

Statistical analyses were performed using SAS University Edition (SAS Institute Inc.). The data were analyzed using one‐way ANOVA with stress type (control, tube‐restraint stress, and film‐restraint stress) as a between‐subjects variable or two‐way repeated measures ANOVA with stress type as a between‐subjects variable and with time as a within‐subject variable. We defined “study‐wide significance” as statistical significance that survived the Benjamini‐Hochberg false discovery rate (FDR) correction^19^, ^20^ for controlling for multiple hypothesis testing based on the number of behavioral measures of the test battery. “Nominal significance” was defined as having achieved statistical significance without the FDR correction (uncorrected *P* < .05). When significant interactions between the two factors were observed, simple interactions and simple main effects were examined, as appropriate. Post hoc multiple comparisons were further performed using Fisher's LSD with Bonferroni correction for multiple comparisons. All of the *P*‐values are presented as unadjusted values. Values in graphs are expressed as the mean ± SEM.

## 3 RESULTS

### 3.1 Decreased body weight, slightly higher locomotor activity, and slightly altered depression‐related behaviors were observed in mice subjected to restraint stress for 10 days

**Figure 2:**
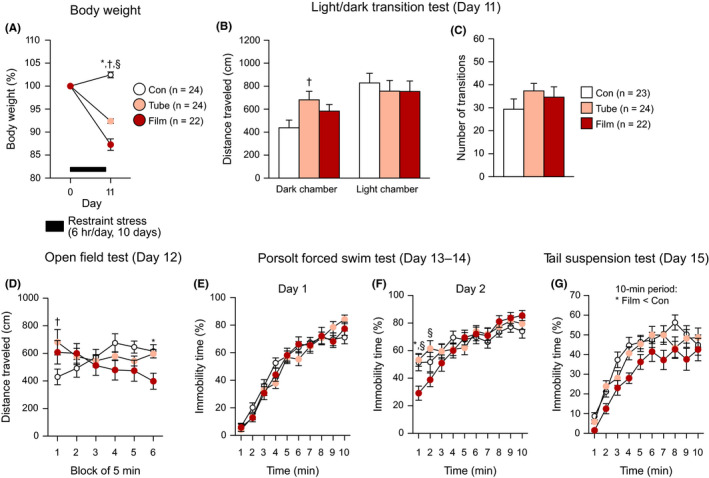
Decreased body weights, increased locomotor activity, and altered depression‐related behaviors in mice exposed to restraint stress for 10 d. The mice exposed to restraint stress for 10 d were assessed for body weight, locomotor activity, and anxiety‐like and depression‐related behaviors. A, Body weights before and after the 10‐d stress session. B, C, Light/dark transition test: (B) distance traveled (cm) in the dark and light chambers, (C) number of transitions between the chambers. D, Distance traveled (cm) in the open field test. E, F, Porsolt forced swim test: percentage of immobility time (%) on test Day 1 (E) and on test Day 2 (F). G, Percentage of immobility time (%) in the tail suspension test. Values are the means ± SEM. When ANOVA indicated a nominally significant effect (*P* < .05), group differences were analyzed using Bonferroni's multiple comparisons test: *P < .05 (Film vs Con), ^†^ *P* < .05 (Tube vs Con), ^§^ *P* < .05 (Film vs Tube)

For body weight, two‐way repeated measures ANOVA showed that there was a significant main effect of stress (*F*_2,67_ = 86.13, *P* < .0001) and a significant stress × day interaction (*F*_2,67_ = 86.13, *P* < .0001) in the first cohort of 10‐day restraint‐stressed mice and nonstressed control mice (Figure 2A). On the day after the 10‐day stress session, film‐ and tube‐restraint–stressed mice had lower body weights than control mice (*P* < .0001 and *P* < .0001), and body weights were lower in film‐restraint–stressed mice than in tube‐restraint–stressed mice (*P* < .0001).

In the light/dark transition test, the behavioral data from one nonstressed control mouse were excluded from the analysis due to a technical problem (video images were not recorded by the image analyzing system). The statistical analysis revealed that there were nominally significant main effects of stress on the distance traveled in the dark chamber (Figure 2B, left: *F*_2,66_ = 3.40, *P* = .0394) and time spent in the light chamber (Figure S1A: *F*_2,66_ = 3.71, *P* = .0297). Tube‐restraint–stressed mice traveled a longer distance in the dark chamber (*P* = .0116) and spent less time in the light chamber (*P* = .0152) than control mice. Film‐restraint–stressed mice also spent less time in the light chamber than control mice, although the difference did not reach significance after Bonferroni correction (*P* = .0314). There were no significant effects of stress on the distance traveled in the light chamber, the number of transitions, or latency to first enter the light chamber (Figure 2B,C and Figure S1B: for the statistical results, see Table S1).

Increased locomotor activity in the stressed mice was also observed in the open field test during the first 5‐minute block, as indicated by increases in distance traveled (Figure 2D: stress effect, *F*_2,67_ = 0.72, *P* = .4926; stress × time interaction, *F*_10,335_ = 4.61, *P* < .0001) and stereotypic counts (Figure S1C: stress effect, *F*_2,67_ = 2.16, *P* = .1230; stress × time interaction, *F*_10,335_ = 2.26, *P* = .0145). The post hoc comparisons revealed that tube‐restraint–stressed mice traveled a longer distance and showed more stereotypic behavior than control mice (*P* = .0033 and *P* = .0003, respectively), and similarly, film‐restraint–stressed mice tended to travel a longer distance and displayed more stereotypic behavior than control mice (*P* = .0411 and *P* = .0007). In contrast, during the last 5‐minute block, film‐restraint–stressed mice traveled a shorter distance than the other groups of mice (vs Con, *P* = .0129; vs Tube, *P* = .0228). There was no significant main effect of stress and no significant stress × time interaction on vertical activity or time spent in the center (Figure S1D,E: for statistical results, see Table S1).

In the Porsolt forced swim test, there were no significant main effects or interactions for immobility (Figure 2E) or distance traveled (Figure S1F) on test Day 1 (Table S1). On test Day 2, significant stress × time interactions were found in measures of immobility (Figure 2F: stress effect, *F*_2,67_ = 0.65, *P* = .5276; stress × time interaction, *F*_18,603_ = 2.26, *P* = .0022) and distance traveled (Figure S1G: stress effect, *F*_2,67_ = 0.47, *P* = .6280; stress × time interaction, *F*_18,603_ = 2.37, *P* = .0012). Film‐restraint–stressed mice showed less immobility and longer distance traveled than control and tube‐restraint–stressed mice during the first 2 minutes of the test (for immobility in the first min, film < Con and Tube, *P* = .0015 and *P* = .0006; for immobility in the second min, film < Con and Tube, *P* = .0652 and *P* = .0012; for distance traveled in the first min, film > Con and Tube, *P* = .0289 and *P* = .0054). During the tenth minute of the test, film‐restraint–stressed mice traveled shorter distances than control mice (*P* = .0116).

In the tail suspension test, there was a nominally significant effect of stress (Figure 2G: stress effect, *F*_2,67_ = 5.34, *P* = .0070; stress × time interaction, *F*_18,603_ = 0.90, *P* = .5780). The post hoc comparisons revealed that film‐restraint–stressed mice exhibited less immobility than control mice (*P* = .0026) and showed a trend toward decreased immobility compared with tube‐restraint–stressed mice, although this result did not reach significance after Bonferroni correction (*P* = .0177).

Regarding the sucrose preference test, there was no significant effect of stress on the percentage of sucrose preference (Figure S1H and Table S1).

### 3.2 Decreased body weight, heightened locomotor activity, and altered depression‐related behaviors were observed in mice subjected to restraint stress for 21 days

**Figure 3:**
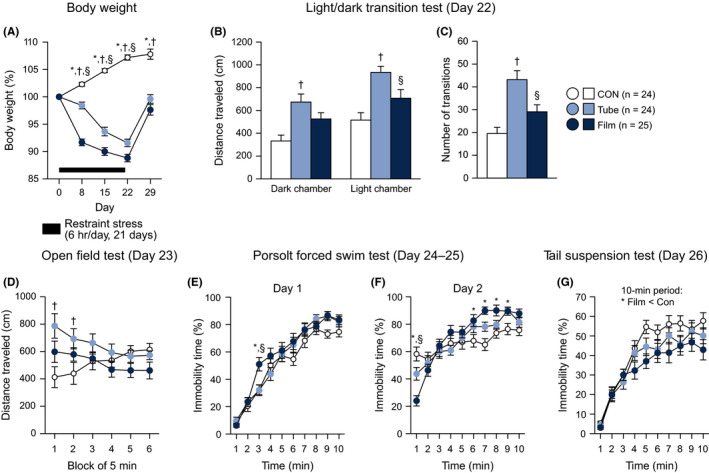
Decreased body weights, increased locomotor activity, and altered depression‐related behaviors in mice exposed to restraint stress for 21 d. The mice exposed to restraint stress for 21 d were assessed for weekly body weights, locomotor activity, and anxiety‐like and depression‐related behaviors. A, Body weights were measured weekly. B, C, Light/dark transition test: (B) distance traveled (cm) in the dark and light chambers, (C) number of transitions between the chambers. D, Distance traveled (cm) in the open field test. E, F, Porsolt forced swim test: percentage of immobility time (%) on test Day 1 (E) and on test Day 2 (F). G, Percentage of immobility time (%) in the tail suspension test. Values are the means ± SEM. When ANOVA indicated a nominally significant effect (*P* < .05), group differences were analyzed using Bonferroni's multiple comparisons test: *P < .05 (Film vs Con), ^†^ *P* < .05 (Tube vs Con), ^§^ *P* < .05 (Film vs Tube)

In the second cohort of mice, body weights were measured weekly during the 3‐week stress session and again 1 week after the stress sessions had ended (Figure 3A). There was a significant main effect of stress and a significant stress × day interaction on body weight (stress effect, *F*_2,70_ = 164.17, *P* < .0001; stress × day interaction, *F*_8,280_ = 61.48, *P* < .0001). After 1 week of stress exposure, film‐ and tube‐restraint–stressed mice had lower body weights than nonstressed control mice (on Days 8, 15, 22, and 29; all *P* < .0001). On Days 8‐22, the body weights were lower in film‐restraint–stressed mice than in tube‐restraint–stressed mice (for Day 8, *P* < .0001; for Day 15, *P* < .0001; for Day 22, *P* = .0017), while no significant differences were found between the two stressed groups of mice on Days 29 after Bonferroni correction (*P* = .0242).

In the light/dark transition test, conducted one day after the 21‐day stress session had ended, there were significant effects of stress on the distance traveled in the dark chamber (Figure 3B, left: *F*_2,70_ = 8.25, *P* = .0006), distance traveled in the light chamber (Figure 3B, right: *F*_2,70_ = 10.14, *P* = .0001), and number of transitions (Figure 3C: *F*_2,70_ = 13.07, *P* < .0001). Tube‐ and film‐restraint–stressed mice traveled longer distances in the dark and light chambers and exhibited more transitions than control mice, although the differences between film‐restraint–stressed mice and control mice did not reach significance after Bonferroni correction for any behavioral measures (for distance traveled in the dark chamber, Tube and Film > Con, *P* = .0001 and *P* = .0237; for distance traveled in the light chamber, Tube and Film > Con, *P* < .0001 and *P* = .0408; and for number of transitions, Tube and Film > Con, *P* < .0001 and *P* = .0419). Additionally, a longer distance traveled in the light chamber and a higher number of transitions were observed in tube‐restraint–stressed mice than in film‐restraint–stressed mice (*P* = .0163 and *P* = .0031, respectively). There was no significant effect of stress on the time spent in the light chamber (Figure S2A: *F*_2,70_ = 0.78, *P* = .4607). A nominally significant effect of stress was found in the latency to enter the light chamber (Figure S2B: *F*_2,70_ = 3.23, *P* = .0456), with a decreased latency to enter the light chamber in tube‐restraint–stressed mice compared with control mice (*P* = .0136).

In the open field test, there were significant stress × time interactions on distance traveled (Figure 3D: stress effect, *F*_2,70_ = 2.35, *P* = .1031; stress × time interaction, *F*_10,350_ = 4.56, *P* < .0001) and stereotypic counts (Figure S2C: stress effect, *F*_2,70_ = 3.22, *P* = .0457; stress × time interaction, *F*_10,350_ = 2.62, *P* = .0043). Tube‐restraint–stressed mice traveled longer distances than control mice in the first and second 5‐minute blocks of the test (*P* < .0001 and *P* = .0036, respectively), and film‐restraint stress mice also exhibited longer distances traveled than control mice in the first block, although the result failed to reach significance after Bonferroni correction (*P* = .0306). Tube‐restraint–stressed mice showed a tendency toward an increase in the distance traveled compared with film‐restraint–stressed mice in the first block (*P* = .0274). Similarly, tube‐ and film‐restraint–stressed mice showed more stereotypic behavior than control mice during the first two blocks of the test (Figure S2C: for the first block, Tube and Film > Con, *P* < .0001 and *P* = .0063; for the second block, Tube and Film > Con, *P* = .0108 and *P* = .0091). There were no significant effects of stress on vertical activity (Figure S2D) or center time (Figure S2E).

In the Porsolt forced swim test, on test Day 1, there was a nominally significant stress × time interaction on immobility time (Figure 3E: stress effect, *F*_2,70_ = 2.71, *P* = .0738; stress × time interaction, *F*_18,630_ = 1.65, *P* = .0443), while there was no significant main effect of stress or interaction on distance traveled (Figure S2F: for statistical results, see Table S2). In the third minute of the test, film‐restraint–stressed mice showed higher levels of immobility than control and tube‐restraint–stressed mice (*P* = .0015 and *P* = .0013, respectively). In the ninth minute of the test, film‐ and tube‐restraint–stressed mice exhibited higher levels of immobility than control mice, although the results did not reach significance after Bonferroni correction (*P* = .0211 and *P* = .0284, respectively). On test Day 2, significant stress × time interactions were found for immobility time (Figure 3F: stress effect, *F*_2,70_ = 2.22, *P* = .1166; stress × time interaction, *F*_18,630_ = 4.64, *P* < .0001) and distance traveled (Figure S2G: stress effect, *F*_2,70_ = 3.85, *P* = .0260; stress × time interaction, *F*_18,630_ = 3.56, *P* < .0001). In the first minute of the test, film‐restraint–stressed mice showed lower levels of immobility and traveled longer distances than the other two groups of mice (for immobility, Film < Con and Tube, *P* < .0001 and *P* = .0011; for distance traveled, Film > Con and Tube, *P* < .0001 and *P* = .0190), and tube‐restraint–stressed mice also tended to display lower levels of immobility than control mice (*P* = .0177). In contrast to the first minute of test, film‐ and tube‐restraint–stressed mice exhibited higher levels of immobility than control mice during the latter half of the testing period (for 6 minutes, Film and Tube > Con, *P* = .0146 and *P* = .0897; for 7 minutes, Film and Tube > Con, *P* < .0001 and *P* = .0315; for 8 minutes, Film and Tube > Con, *P* = .0065 and *P* = .3240; for 9 minutes, Film and Tube > Con, *P* = .0304 and *P* = .0232). Additionally, film‐ and tube‐restraint–stressed mice traveled shorter distances than control mice (for 6 minutes, Film and Tube < Con, *P* = .0018 and *P* = .0449; for 7 minutes, Film and Tube < Con, *P* = .0002 and *P* = .0757; for 8 minutes, Film and Tube < Con, *P* = .0116 and *P* = .1939; for 9 minutes, Film and Tube < Con, *P* = .0355 and *P* = .0077).

In the tail suspension test (Figure 3G), there was a significant main effect of stress on the percentage of immobility time (stress effect, *F*_2,70_ = 4.89, *P* = .0103; stress × time interaction, *F*_18,630_ = 0.97, *P* = .4940). Post hoc comparisons revealed that film‐restraint–stressed mice were immobile for a significantly shorter amount of time than control mice (*P* = .0027), and no significant differences were found between the other groups.

There was no significant effect of stress on the percentage of sucrose preference tested on Days 26‐28 (Figure S2H: *F*_2,70_ = 1.54, *P* = .2210).

### 3.3 Plasma corticosterone levels in tube‐ and film‐restraint–stressed mice

**Figure 4:**
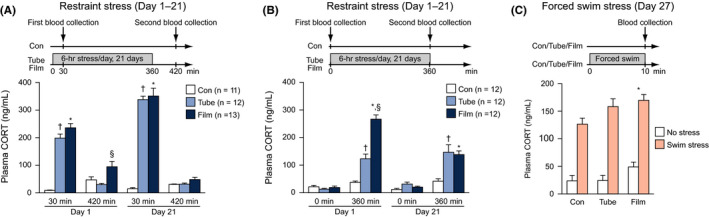
Plasma corticosterone levels of restraint‐stressed mice. Plasma corticosterone levels were measured at two time points during and after the 6‐h stress period on Days 1 and 21. A, On Days 1 and 21, blood samples were collected from half of the cohort of mice 30 min after the start of stress exposure (30 min) and again 60 min after the termination of the 6‐h stress exposure (420 min). B, Similarly, in the other half of the cohort, blood collection was performed immediately before and after stress exposure (0 and 360 min) on Days 1 and 21. C, Some of the mice exposed to restraint stress for 21 d and the control mice of the nonstressed group were subjected to the forced swim test for 10 min on day 27 (Con, n = 6; Tube, n = 6; Film, n = 8). Immediately after the test, blood was collected from the stressed mice and the mice that had no prior experience with the forced swim test (Con, n = 5; Tube, n = 6; Film, n = 8). Values are the means ± SEM. When ANOVA indicated a nominally significant effect (*P* < .05), group differences were analyzed using Bonferroni's multiple comparisons test: *P < .05 (Film vs Con), ^†^ *P* < .05 (Tube vs Con), ^§^ *P* < .05 (Film vs Tube)

In the third cohort of mice subjected to restraint stress for 21 days, plasma corticosterone levels were measured at different time points on Days 1 and 21 of the stress procedure. In half of the cohort, blood was collected 30 minutes after stress exposure (30 minutes) and 60 minutes after the termination of the 6‐hour stress session (420 minutes) to examine the acute effects of stress on the HPA system and to evaluate the negative feedback function. There was a significant main effect of stress (*F*_2,33_ = 114.17, *P* < .0001) and a significant stress × time interaction (*F*_6,99_ = 44.99, *P* < .0001) on plasma corticosterone levels (Figure 4A). On Day 1, higher corticosterone levels were found in film‐ and tube‐restraint–stressed mice than in nonstressed control mice (Film and Tube > Con, all *P* < .0001; Film > Tube, *P* = .0531). On Day 1, higher corticosterone levels were also observed 60 minutes after the 6‐hour stress session in the film‐restraint–stressed mice than in the other groups of mice (Film > Con and Tube, *P* = .0169 and *P* = .0010), although the difference between film‐restraint–stressed mice and control mice did not reach significance after Bonferroni correction. The stress‐induced increase in corticosterone levels after 30 minutes of stress exposure on Day 21 (Film and Tube > Con, all *P* < .0001) was higher than that measured on Day 1 (for Film, *P* < .0001; for Tube, *P* < .0001). The corticosterone levels in the two stressed groups of mice did not differ at any time point on Day 21.

In the other half of the cohort, blood collections were performed before the start of the 6‐hour stress session (0 minute) and immediately after the 6‐hour stress session (360 minutes) on Days 1 and 21 to examine basal levels of corticosterone and the effects of prolonged stress on corticosterone levels. There was a significant main effect of stress (*F*_2,33_ = 30.45, *P* < .0001) and a significant stress × time interaction (*F*_6,99_ = 29.94, *P* < .0001) on plasma corticosterone levels (Figure 4B). There were no significant differences in basal corticosterone levels at 0 minute among the three groups of mice on Days 1 or 21. After a 6‐hour stress session, higher corticosterone levels were found in the two stressed groups of mice than in nonstressed control mice on Days 1 and 21 (all *P* < .0001): the film‐restraint–stressed mice showed higher corticosterone levels than tube‐restraint–stressed mice on Day 1 (*P* < .0001). The corticosterone level after 360 minutes of stress exposure on Day 21 was lower than that measured on Day 1 in the film‐restraint–stressed mice (*P* < .0001), and the difference between the two stressed groups was not observed on Day 21 (*P* = .6230).

Similar to the second cohort of mice, film‐ and tube‐restraint–stressed mice in the third cohort had lower body weights than control mice from Day 8 to Day 22 (Figure S3A: stress effect, *F*_2,69_ = 119.20, *P* < .0001; stress × day interaction, *F*_6,207_ = 71.32, *P* < .0001; for Days 8, 15, and 22, Film and Tube < Con, all *P* < .0001). In addition, body weights of film‐restraint–stressed mice were lower than those of tube‐restraint–stressed mice on Days 8, 15, and 22 (*P* < .0002, *P* = .0003, and *P* = .0002, respectively).

In the sucrose preference test, starting one day after the 21‐day stress session (Figure S3B), film‐restraint–stressed mice showed less sucrose preference than control mice (Film < Con, *P* = .0133; Tube < Con, *P* = .1348), although the main effect of stress in ANOVA did not reach study‐wide significance (*F*_2,36_ = 3.40, *P* = .0445). During the last 2 days of the test, the three groups of mice did not differ in sucrose preference (*F*_2,36_ = 0.08, *P* = .9274).

On the day following the sucrose preference test, half of the mice in each group were subjected to the forced swim test for 10 minutes and half were left undisturbed in their home cages, and then blood samples were collected to further evaluate corticosterone levels in response to a novel stressful environment or forced swim stress. During the forced swim test, film‐ and tube‐restraint–stressed mice exhibited higher levels of immobility (Figure S3C: stress effect, *F*_2,17_ = 5.80, *P* = .0120; stress × time interaction, *F*_18,153_ = 0.95, *P* = .5231; Film and Tube > Con, *P* = .0031 and *P* = .0127) and traveled shorter distances (Figure S3D: stress, *F*_2,17_ = 5.29, *P* = .0164; stress × time interaction, *F*_18,153_ = 1.36, *P* = .1574; Film and Tube < Con, *P* = .0066 and *P* = .0229) than control mice. Immediately after the test, film‐restraint–stressed mice had higher levels of corticosterone than control mice (Figure 4C: stress effect, *F*_2,33_ = 5.17, *P* = .0111; stress × time interaction, *F*_2,33_ = 0.94, *P* = .4022; Film > Con, *P* = .0031; Tube > Con, *P* = .1662; Film > Tube, *P* = .0937).

## 4 DISCUSSION

In this study, we evaluated the effects of repeated exposure to restraint stress for 10 or 21 days using two types of restraint apparatuses, a well‐ventilated 50‐mL plastic conical tube and a tapered plastic film envelope, on body weights, locomotor activity, anxiety‐like behavior, depression‐related behavior, and plasma corticosterone levels in inbred male BALB/cAJcl mice. Our results showed that film‐restraint stress had more pronounced effects on body weight and depression‐related behavior than tube‐restraint stress, whereas tube‐restraint stress markedly increased locomotor activity and caused moderate weight loss and a relatively slight change in depression‐related behaviors. Film‐restraint stress induced higher plasma corticosterone levels than tube‐restraint stress when measured on the first day of the repeated stress procedure. Additionally, increased corticosterone levels in response to a novel stressful condition or forced swim stress were observed in film‐restraint–stressed mice compared with those in control mice, while such significant differences were not found between tube‐restraint–stressed mice and control mice. These results demonstrate that film‐restraint stress has more substantial effects on body weight, depression‐related behavior, and corticosterone levels than tube‐restraint stress in adult male BALB/cAJcl mice.

Body weight changes are a frequently observed phenotype following chronic restraint stress.^7^, ^21^, ^22^ Our data showed that repeated exposure to restraint stress in a film envelope caused a larger decrease in body weight than repeated restraint stress by tube at least 1 week after the beginning of the repeated stress. These results were replicated in another experiment in an independent cohort of mice, indicating that the results are robust and that stress caused by different restraint apparatuses can have substantially different effects on body weights. These data suggest that restraint using a film envelope induces a higher intensity of stress than restraint using a tube. Such differential stress intensities may have been found because the film envelope was not ventilated, except for a hole that allowed breathing from the nose, and it was twisted and squeezed to severely restrict movement, whereas the tube was well‐ventilated and allowed limited room for movement. However, no significant differences in the body weights between the two restraint stress groups were observed 1 week after the termination of the stress procedure, suggesting that the differential effects of stress caused by the two restraint apparatuses did not last long.

Film‐restraint–stressed mice showed higher corticosterone levels than tube‐restraint–stressed mice immediately after and 60 minutes after the termination of the 6‐hour stress session on the 1st day of the repeated stress procedure but not after the 21st day of the procedure. These results, which are consistent with the observations for body weight changes, indicate that film‐restraint stress has a more significant impact on the neuroendocrine response than tube‐restraint stress, at least during the early period of stress exposure. The markedly heightened neuroendocrine response in film‐restraint–stressed mice might be attenuated until the termination of 6‐hour stress exposure on Day 21, possibly due to the mechanism that enables organisms to maintain homeostasis or to habituate to repeated stress.^23^, ^24^, ^25^, ^26^, ^27^ Together, these data suggest that repeated exposure to restraint stress enhances the endocrine response to stress, with more marked changes being seen after film‐restraint stress than after tube‐restraint stress.

Restraint stress with different durations (several minutes to 6 or more hours) and frequencies (1 day to 3 weeks) has been reported to cause various behavioral changes, such as altered locomotor activity, increased anxiety‐like behavior, and impairments in learning and memory.^6^, ^28^, ^29^, ^30^, ^31^ The present results showed that 10‐day stress exposure caused slight, but significant, behavioral changes: tube‐restraint–stressed mice traveled longer distances in the open field and light/dark transition tests than nonstressed mice. Despite their increased locomotor activity, 10‐day tube‐restraint–stressed mice showed no differences in immobility or distance traveled in the forced swim and tail suspension tests compared to nonstressed mice. Film‐restraint–stressed mice showed more locomotor activity during the early period of the open field test and less immobility in the early period of the forced swim test and in the tail suspension test than nonstressed mice. These results indicate that 10 days of exposure to film‐restraint stress, but not tube‐restraint stress, has significant effects on depression‐related behaviors. More pronounced effects on behavior were observed in mice exposed to 21 days in either stress protocol. An increase in locomotor activity was observed in tube‐restraint–stressed mice, which was higher than that seen in film‐restraint–stressed mice. In the forced swim test, immobility was increased in 21‐day restraint‐stressed mice, with higher levels of immobility being seen in film‐restraint–stressed mice than in tube‐restraint–stressed mice or nonstressed mice during the end of the test period.

The sucrose preference test has been extensively used to measure anhedonia when evaluating animal models of depression.^32^, ^33^ When the first cohort of mice was tested 5‐7 days after the termination of repeated stress, film‐ and tube‐restraint–stressed mice showed no differences from nonstressed mice in sucrose preference. However, in another cohort of mice in which the test was conducted for two days after the 21‐day stress procedure, film‐restraint–stressed mice showed less sucrose preference than nonstressed mice, and this effect disappeared after the 2‐day test session. Similar to the findings for the body weights described above, these observations suggest that film‐restraint stress has a larger impact on the behavior, although the effects did not last longer than two days.

In this study, half of the third cohort of mice was subjected to the forced swim test to further evaluate the effects of 21 days of restraint stress on plasma corticosterone levels in response to a novel stressor such as forced swimming. In the third cohort of mice, increased depression‐related behavior was found in the forced swim test, and there was no difference in the level of immobility between the two restraint stress groups, which does not seem to support our conclusion that differential effects on depression‐related behavior can be seen as a result of these two types of restraint stress apparatuses. Although we do not know the exact reason for these inconsistent results, one possibility is that the differential effects of the restraint apparatuses might be masked by the added stress from invasive blood sampling and/or having no prior experience with the behavioral tests. These observations in the third cohort are based on a small number of animals, and further study may be needed to validate the effects of the different restraint apparatuses. Regardless, the results from the two independent cohorts of 21‐day restraint‐stressed mice strengthen the conclusion that 21 days of restraint stress results in increased depression‐related behavior in the forced swim paradigm and that film‐restraint stress is more intense than tube‐restraint stress, in terms of the extent of body weight loss.

In this study, we found that, in general, film‐restraint stress has more substantial effects on blood corticosterone level, body weight, and depression‐related behavior than tube‐restraint stress, although the behavioral differences between the two stress protocols might have been influenced by heightened locomotor activity and unknown confounding factors. These findings can help guide which restraint stress procedures should be used, depending on the objectives of a given study, to generate animal models of stress‐induced neuropsychiatric disorders.

## CONFLICT OF INTEREST

The authors declare no conflict of interests.

## AUTHOR CONTRIBUTIONS

HS designed and performed the experiments, analyzed the data, and wrote manuscript. TM coordinated the study and interpreted the data.

## DATA REPOSITORY

All the data directly associated with the results of this study are included in the Data S1. The data from the behavioral tests are also accessible through the online database “Mouse Phenotype Database” (http://www.mouse-phenotype.org/).

## ANIMAL STUDIES

All of the experimental procedures were approved by the Institutional Animal Care and Use Committee of Fujita Health University.
